# Retinal changes in COVID-19 hospitalized cases

**DOI:** 10.1371/journal.pone.0243346

**Published:** 2020-12-03

**Authors:** Rafael Lani-Louzada, Carolina do Val Ferreira Ramos, Ricardo Mello Cordeiro, Alfredo A. Sadun

**Affiliations:** 1 Hospital Nossa Senhora da Saúde, Santa Casa da Misericórdia do Rio de Janeiro (Hospital da Gamboa), Instituto de Oftalmologia do Rio de Janeiro, Rio de Janeiro, Rio de Janeiro, Brazil; 2 Clínica Nossa Senhora da Paz, Rio de Janeiro, Rio de Janeiro, Brazil; 3 Departamento de Clínica Médica, Hospital de Clínicas de Jacarepaguá, Rio de Janeiro, Rio de Janeiro, Brazil; 4 Departamento de Clínica Médica, Hospital de Clínicas Mário Lioni, Rio de Janeiro, Rio de Janeiro, Brazil; 5 Department of Ophthalmology, David Geffen School of Medicine at UCLA, Doheny Eye Institute, Los Angeles, California, United States of America; University of Florida, UNITED STATES

## Abstract

The main objective of this study was to evaluate the retinas of severely or critically ill COVID-19 patients during their hospital stay, at varying time points after symptoms onset. This was a case series observed during May 2020 in two referral centers for COVID-19 treatment in Rio de Janeiro, Brazil. 47 eyes from 25 hospitalized patients with severe or critical confirmed illness were evaluated. A handheld retinal camera was used to acquire bilateral fundus images at several time points after symptoms onset. Electronic health records were retrospectively analyzed and clinical data collected. Severe and critical diseases were noticed in 52% (13/25) and 48% (12/25) of enrolled patients, respectively. Retinal changes were present in 12% (3/25) of patients: a 35 year-old male demonstrated bilateral nerve fiber layer infarcts and microhemorrhages in the papillomacular bundle, but required mechanical ventilation and developed severe anemia and systemic hypotension, acute kidney injury and neurologic symptoms during the course of the disease (critical illness); a 56 year-old male, who required full enoxaparin anticoagulation due to particularly elevated D-dimer (>5.0 mcg/mL), demonstrated unilateral and isolated flame-shaped hemorrhages; and a 49 year-old hypertensive male showed bilateral and discrete retinal dot and blot microhemorrhages. The other 22 patients evaluated did not demonstrate convincing retinal changes upon examination. There was no correlation between disease severity and admission serum levels of CRP, D-dimer and ferritin. This was the first study to show that vascular retinal changes may be present in not insignificant numbers of severe or critical COVID-19 inpatients. These retinal changes, only seen after morbid developments, were likely secondary to clinical intercurrences or comorbidities instead of a direct damage by SARS-CoV-2, and may be important and easily accessible outcome measures of therapeutic interventions and sentinels of neurologic and systemic diseases during COVID-19 pandemic.

## Introduction

The novel coronavirus disease in 2019 (COVID-19) has been responsible for over 16 million confirmed cases worldwide, with more than 800,000 confirmed deaths [1]. The respiratory symptoms that characterize COVID-19 [2, 3] may be associated with a wide range of neurological complications, including cerebrovascular diseases (acute ischemic infarcts in small or large arterial vessels, cerebral venous sinus thrombosis and intracranial hemorrhage), usually related to severe or critical disease phenotypes, and which may significantly contribute to patients morbidity and mortality [4]. However, the pathophysiology behind COVID-19 associated brain damage is still poorly understood [5], and both a direct viral neurotropism or indirect mechanisms involving dysregulated inflammatory cytokines release (“cytokine storm”), vascular endothelial injury and hypercoagulation cascades triggering/ disseminated intravascular coagulation are under investigation [4, 6].

Genomic and structural analyses have reported that one of the major players for SARS-CoV-2 entry into the host cell is the cell surface enzyme protein angiotensin-converting enzyme 2 (ACE2), which acts as the functional receptor for SARS-CoV-2 to bind to host cells, followed by cellular transmembrane protease serine 2 (TMPRSS2) for priming [7, 8]. In humans, ACE2 is present in the CNS, lungs, kidney, enterocytes, nose, liver, immune system and blood vessels [9, 10]. In the human eye, this protein has been observed in cornea, conjunctiva [11], aqueous humor [12] and retina [13]. Indeed, evidence of an intraocular renin-angiotensin system regulating important aspects of ocular physiology (e.g. retinal vascular tone and aqueous dynamic) has been demonstrated [12, 14], and viral ribonucleic acid has been detected by reverse transcription polymerase chain reaction (RT-PCR) in retinal biopsies of deceased COVID-19 patients [15], which together endorses the rationale for an ophthalmological syndrome of COVID-19, with some pathophysiology still unknown.

John Dowling referred to the human retina as “the approachable part of the brain” [16], and it’s complex neuronal and vascular milieu can be clinically visualized with proper instruments. Thus, a comprehensive depiction of the ocular fundus from hospitalized SARS-COV-2 patients, with consideration of distinct disease stages and severity, may lead to further insights on the CNS pathology associated with this novel and challenging disease.

The present study was designed to evaluate the retinas of confirmed COVID-19 patients admitted to hospital units for clinical stabilization and oxygen supplementation, presenting with severe or critical disease, at varying time points after symptoms onset and to measure these changes in reference to interventions and complications.

## Materials and methods

### Study oversight

A transversal and observational study was conducted during May 2020 in accordance with the tenets of the Declaration of Helsinki and with ethics approval by the Committee of Ethics in Research from Amil Clinical Research board. Patients from two referral centers for COVID-19 treatment in Rio de Janeiro, Brazil, were recruited and gave written informed consent before fundus examination.

Study inclusion criteria included subjects hospitalized for clinical instability caused by COVID-19 who were able to provide informed consent, or with a legal representative able to provide informed consent in the name of the participant. Participants were confirmed cases of COVID-19, which was defined as a positive result on reverse-transcriptase polymerase chain reaction (RT-PCR) assay of nasal swab specimens. Exclusion criteria were patients with negative RT-PCR for SARS-CoV-2 on nasal swab specimens, and patients with previously diagnosed retinal diseases such as moderate/severe diabetic retinopathy or optic neuropathies.

### Participants and study procedures

In total, 47 eyes from 25 inpatients from two referral centers for COVID-19 treatment in Rio de Janeiro, Brazil, were enrolled in the study. Electronic health records were retrospectively analyzed and clinical data collected including age, gender, symptoms and day after symptoms onset for hospitalization, admission serous levels of D-dimer, ferritin and C-reactive protein (CRP), day after symptoms onset ophthalmological examination, extension of ground-glass opacities on chest CT and systemic comorbidities.

Cases were classified as severe or critical according to the Diagnosis and Treatment Protocol for Novel Coronavirus Pneumonia Trial Version 7, released by the National Health Commission & National Administration of Traditional Chinese Medicine on March 3rd, 2020 [17]. Briefly, severe cases included patients with radiological findings of viral pneumonia and clear respiratory distress, characterized by respiratory rate ≥ 30 breaths/minute, or oxygen saturation ≤ 93% at rest; critical cases were those requiring continuous monitoring in intensive care units (ICU) for the eminent risk of hypoxemia and respiratory failure, as well as patients with indications for invasive mechanical ventilation, diagnosis of shock or any other organ failure requiring ICU care.

Ocular fundi were evaluated by using a handheld digital retinal camera connected with a smartphone, with nine internal points of gaze fixation for central and peripheral retinal imaging (Phelcom Eyer; Phelcom Technologies, Sao Paulo, Brazil). The procedure was performed in a bedside manner. The examiner (RL) wore proper personal protective equipment including inner and outer gloves, fit-tested N95 mask plus medical mask, gown and goggle [18]. For better retinal documentation, most patients had their pupils pharmacologically dilated by topical instillation of tropicamide 10 mg/mL eye drops.

The retinal camera automatically registered both colored and red-free retinographies in each acquisition. Retinal images were sent to a cloud server (EyerCloud; Phelcom Technologies, Sao Paulo, Brazil) and independently analyzed by two researchers (RL and CVFR), who were masked as to the patients’ blood parameters and medical history.

### Statistical analysis

Statistical analysis was performed using GraphPad Prism 6.0c software. Data are expressed as mean ± STD. Unpaired two-tailed T-Student test was done to compare the blood parameters mean values of severe and critical disease groups, as well as the mean age and days post symptoms onset for hospital admission and fundus examination. Two-tailed Fisher’s exact test was done to compare proportions (gender, symptoms, past medical history and retinal changes). *P*-values less than 0.05 were considered statistically significant.

## Results

### Demographic and clinical characteristics

Among participants, 64% were men, 36% were women, varying in age between 35 and 75 years (mean ± STD: 51.2 ± 10.8 years). All patients tested positive for SARS-CoV-2 on RT-PCR performed on nasal swab specimens. Regarding disease severity, since all patients required hospitalization for clinical stability, no patients enrolled in the study was classified with mild disease (Table 1). Also, no patient classified as moderate since oxygen supplementation was necessary to all patients in order to maintain normal oxygen levels. Severe disease was noticed in 13 out of 25 patients (52%) during ophthalmic examination. Finally, 48% patients in our study (12/25) presented critical disease requiring ICU monitorization at some moment during their hospital stay (Table 1). Among them, 67% (8/12) needed mechanical ventilation through an endotracheal tube or tracheostomy, and 33% (4/12) required high-flow oxygen therapy (9–10L/min) delivered using a Venturi mask.

**Table 1:** General and demographic characteristics from enrolled population.

Parameter	Total	Disease phenotype		*P-*value (severe vs critical)
		Severe	Critical	
Percentage (number)		52% (13/25)	48% (12/25)	NA
Age (years)	35–75	38–75	35–67	0.1091
	(51.2 ± 10.8)*	(54.5 ± 10.0)*	(47.5 ± 10.9)*	
Gender				1.0000
Male (%)	64% (16/25)	61.5% (8/13)	66.7% (8/12)	
Female (%)	36% (9/25)	38.5% (5/13)	33.3% (4/12)	
Symptoms				
Dyspnea	88% (22/25)	84.6% (11/13)	91.6% (11/12)	1.0000
Cough	84% (21/25)	76.9% (10/13)	91.6% (11/12)	0.5930
Fever	80% (20/25)	84.6% (11/13)	75.0% (9/12)	0.6447
Myalgia	56% (14/25)	61.3% (8/13)	50.0% (6/12)	0.6951
Fatigue	32% (8/25)	30.8% (4/13)	33.3% (4/12)	1.0000
Headache	24% (6/25)	23.1% (3/13)	25.0% (3/12)	1.0000
Nasal Congestion	16% (4/25)	7.7% (1/13)	25.0% (3/12)	0.3217
Anosmia	8% (2/25)	0% (0/13)	16.7% (2/12)	0.2200
Ageusia	8% (2/25)	0% (0/13)	16.7% (2/12)	0.2200
Nausea	4% (1/25)	7.7% (1/13)	0% (0/12)	1.0000
Odynophagia	4% (1/25)	0% (0/13)	8.3% (1/12)	0.4800
Hyporexia	4% (1/25)	7.7% (1/13)	0% (0/12)	1.0000
Rhinorrhea	4% (1/25)	7.7% (1/13)	0% (0/12)	1.0000
Days post symptoms onset				
Hospital admission	2–14 (8.6 ± 3·0)*	2–14 (8.8 ± 3.5)*	5–14 (8.3 ± 2.6)*	0.6876
Fundus examination	10–64	10–22	14–64	0.0021
	(23.9 ± 14.2)*	(15.3 ± 3.5)*	(33.25 ± 15.6)*	
Past Medical History				
Arterial Hypertension	60% (15/25)	53.9% (7/13)	66.7% (8/12)	0.6882
Diabetes	32% (8/25)	30.8% (4/13)	33.3% (4/12)	1.0000
Obesity	32% (8/25)	23.1% (3/13)	41.7% (5/12)	0.4110
None	24% (6/25)	30.8% (4/13)	16.7% (2/12)	0.6447
Asthma	4% (1/25)	7.7% (1/13)	0% (0/12)	1.0000
Cancer	4% (1/25)	7.7% (1/13)	0% (0/12)	1.0000
COPD	4% (1/25)	0% (0/13)	8.3% (1/12)	0.4800
Hypothyroidism	4% (1/25)	0% (0/13)	8.3% (1/12)	0.4800
Retinal changes	12% (3/25)	15.4% (2/13)	8.3% (1/12)	1.0000

COPD = Chronic Obstructive Pulmonary Disease

*Mean ± STD; NA: Not Applicable.

Among symptoms typically found in COVID-19, we observed dyspnea (88%), cough (84%), fever (80%), myalgia (56%), fatigue (32%), headache (24%), nasal congestion (16%), anosmia (8%), ageusia (8%), nausea (4%), odynophagia (4%), hyporexia (4%) and rhinorrhea (4%) (Table 1).

In terms of pre-existing medical conditions, 60% had arterial hypertension; 32% and 28% were diabetic or obese, respectively; 24% had no comorbidities; asthma, cancer, chronic obstructive pulmonary disease (COPD) and hypothyroidism were present in 4% patients each (Table 1). In this study, COVID-19 patients admitted to the inpatient unit during the acute phase were offered a standard treatment based on intravenous azithromycin, amoxicillin clavulanate and hydroxychloroquine, as well as a single dose of oral ivermectin and daily subcutaneous enoxaparin.

A total of four outcome data were evaluated: the one main outcome was retinal changes suggestive of ongoing pathological process; the other three secondary outcomes were comparisons between the two disease severity groups with regard to admission serum level of c-reactive protein (CRP), D-dimer and ferritin.

### Laboratory and imaging on admission

The mean ± STD of C-reactive protein (CRP) on hospital admission was 12.51 ± 6.94 mg/dL (reference value: <1.0 mg/dL), while D-dimer and ferritin and were 1.53 ± 0.99 mcg/mL (reference value: <0.5 mcg/mL; missing data: 1/25 participant) and 1495 ± 921.74 ng/mL (reference value: 11–307 ng/mL; missing data: 3/25 participants), respectively. When correlated with disease severity, the admission blood parameters analyzed did not show statistically significant differences when comparing patients with severe or critical disease [(CRP–Severe: 10.85 ± 6.74 mg/dL, N = 11 vs Critical: 14.88 ± 7.06 mg/dL, N = 12; p = 0.18); (D-dimer–Severe: 1.43 ± 0.93 mcg/mL, N = 11 vs Critical: 1.70 ± 1.06 mcg/mL, N = 12; p = 0.53); (Ferritin–Severe: 917.00 ± 654.96 ng/mL, N = 11 vs 1677.43 ± 1276.21 ng/mL, N = 10; p = 0.71)].

On chest CT scan, 46% (6/13) of patients with severe disease presented on hospital admission with bilateral ground glass opacities in 25–50% of lung parenchyma, while 15% (2/13) had 50% extension, and 38% (5/13) had >50% of the lung parenchyma affected (missing data: 1/25 participant). Among critical COVID-19 patients, 8% (1/12) presented with 25% of lung parenchyma opacified on admission; 8% (1/12) presented with 25–50% of lung parenchyma with ground glass opacities; 25% (3/12) had 50% ground glass opacities extension in pulmonary chest CT scan; 58% (7/12) had >50% lung compromise; and one patient with critical disease did not have chest CT scan performed on admission to hospital setting. See Table 2 for detailed description of clinical features from each patient enrolled in the study.

**Table 2:** Clinical features individually depicted for each patient.

Patient number	Age (years)	Sex	Symptoms on admission	Hospital admission: days from symptoms onset	Fundus examination: days from symptoms onset	Blood parameter on admission or early hospital stay			Extension of COVID-19 pulmonary findings on chest CT	COVID-19 severity	Complication during hospitalization	Systemic comorbidities
						C-Reactive protein (mg/dL)	D-dimer (mcg/mL)	Ferritin (ng/mL)				
#1	75	Female	Fever, cough, myalgia, dyspnea	7	12	17.7	1.86	1025.0	25–50%	Severe	None	Arterial hypertension; Diabetes Mellitus; Metastatic cancer
#2	47	Female	Fever, cough, headache, nausea, fatigue	10	13	8.7	NA	NA	25–50%	Severe	None	Arterial hypertension; Diabetes Mellitus
#3	48	Male	Cough, nasal congestion, myalgia, dyspnea	14	17	2.2	2.83	1199.0	25–50%	Severe	None	Obesity
#4	50	Male	Fever, cough, myalgia, dyspnea	9	12	15.9	1.34	1845.0	>50%	Severe	None	None
#5	48	Male	Fever, cough, fatigue, dyspnea	8	47	24.1	2.08	>1620.0	>50%	Critical	Respiratory failure requiring mechanical ventilation/ ICU	Arterial hypertension
#6	35	Male	Fever, cough, dyspnea	7	64	6.1	0.27	NA	25–50%	Critical	Respiratory failure requiring mechanical ventilation/ Acute kidney injury/ ICU/ Neuromyopathy of critical illness	Arterial hypertension; Obesity; Hypothyroidism
#7	67	Male	Cough and dyspnea	7	46	15.1	0.55	>1650.0	>50%	Critical	Respiratory failure requiring mechanical ventilation/ Acute kidney injury/ ICU	Arterial hypertension
#8	37	Male	Fever, cough, nasal congestion, myalgia, fatigue, dyspnea	5	18	10.3	1.13	1373.0	25%	Critical	Respiratory failure/ ICU	None
#9	60	Male	Fever, myalgia, fatigue, diarrhea, dyspnea	8	18	16.6	0.7	2558.0	25–50%	Severe	None	Arterial hypertension
#10	63	Male	Fever, cough, myalgia, dyspnea	6	10	10.8	2.15	1346.0	50%	Severe	None	Arterial hypertension; Diabetes Mellitus
#11	43	Male	Fever, cough, headache, dyspnea	9	18	2.8	0.35	1534.0	>50%	Severe	None	Asthma
#12	51	Female	Cough, fatigue, dyspnea, ageusia	9	15	15.8	0.71	280.4	>50%	Critical	Respiratory failure/ ICU	Arterial hypertension; Diabetes Mellitus; Obesity; COPD
#13	57	Male	Fever, fatigue, dyspnea	14	18	22.9	1.0	1993.0	75%	Severe	None	None
#14	38	Female	Cough, fever, rhinorrhea, myalgia, dyspnea	10	18	6.2	0.55	50.86	>50%	Severe	None	None
#15	59	Male	Fever, cough, odynophagia, myalgia,	10	39	8.6	1.38	NA	>50%	Critical	Respiratory failure/ ICU	Diabetes Mellitus
#16	59	Female	Fever, hyporexia, fatigue	5	22	6.8	0.71	592.0	25–50%	Severe	None	Arterial hypertension; Diabetes Mellitus; Obesity
#17	64	Female	Cough, dyspnea	2	13	11.5	1.64	272.0	>50%	Severe	None	Arterial Hypertension; Obesity
#18	56	Male	Fever, cough, headache, myalgia, dyspnea,	13	16	12.1	5.92	1030.0	50%	Severe	None	None
#19	49	Male	Fever, cough, myalgia, dyspnea	8	12	6.8	0.27	1774.0	25–50%	Severe	None	Arterial hypertension
#20	42	Female	Fever, cough, headache, myalgia, fatigue, dyspnea	5	14	9.0	0.93	647.0	50%	Critical	Respiratory failure/ ICU	Arterial hypertension; Obesity
#21	35	Female	Fever, cough, dyspnea	10	47	22.8	3.17	657.0	NA	Critical	Respiratory failure requiring mechanical ventilation/ Acute kidney injury/ ICU	Diabetes Mellitus
#22	62	Male	Fever, cough, nasal congestion, myalgia, anosmia, ageusia, dyspnea	6	34	22.4	2.79	1670.0	50%	Critical	Respiratory failure requiring mechanical ventilation/ acute mesenteric ischemia / ICU	Arterial hypertension; Diabetes Mellitus; Obesity
#23	47	Female	Fever, cough, headache, nasal congestion, myalgia, dyspnea,	14	21	4.1	2.61	555.0	>50%	Critical	Respiratory failure requiring mechanical ventilation/ ICU	Arterial hypertension
#24	37	Male	Fever, headache, myalgia, dyspnea	10	29	20.4	1.4	3769.0	>50%	Critical	Respiratory failure requiring mechanical ventilation/ Acute kidney injury/ Hemorrhagic stroke (subarachnoid and intracerebral hemorrhage)	Arterial hypertension; Obesity
#25	50	Male	Cough, anosmia, dyspnea	8	25	19.8	3.33	3071.0	>50%	Critical	Respiratory failure requiring mechanical ventilation/ ICU	None

NA = Not Applicable

COPD = Chronic Obstructive Pulmonary Disease

### Retinal analysis

Patients were admitted to our hospital facility on average after 8.6 ± 3.0 days of symptoms onset, ranging between 2 to 14 days. The fundus examination was performed from 10 to 64 days after beginning of symptoms, with a mean of 23.9 ± 14.2 days (Tables 1 and 2).

Most patients (21/25) permitted the examination of their retina with pharmacological dilation of the pupils using tropicamide 10 mg/mL eye drop. Both eyes were examined in 23 out of 25 patients. Fundus examination was performed with an Eyer portable retinographer (See Materials and methods Section).

**Fig 1:**
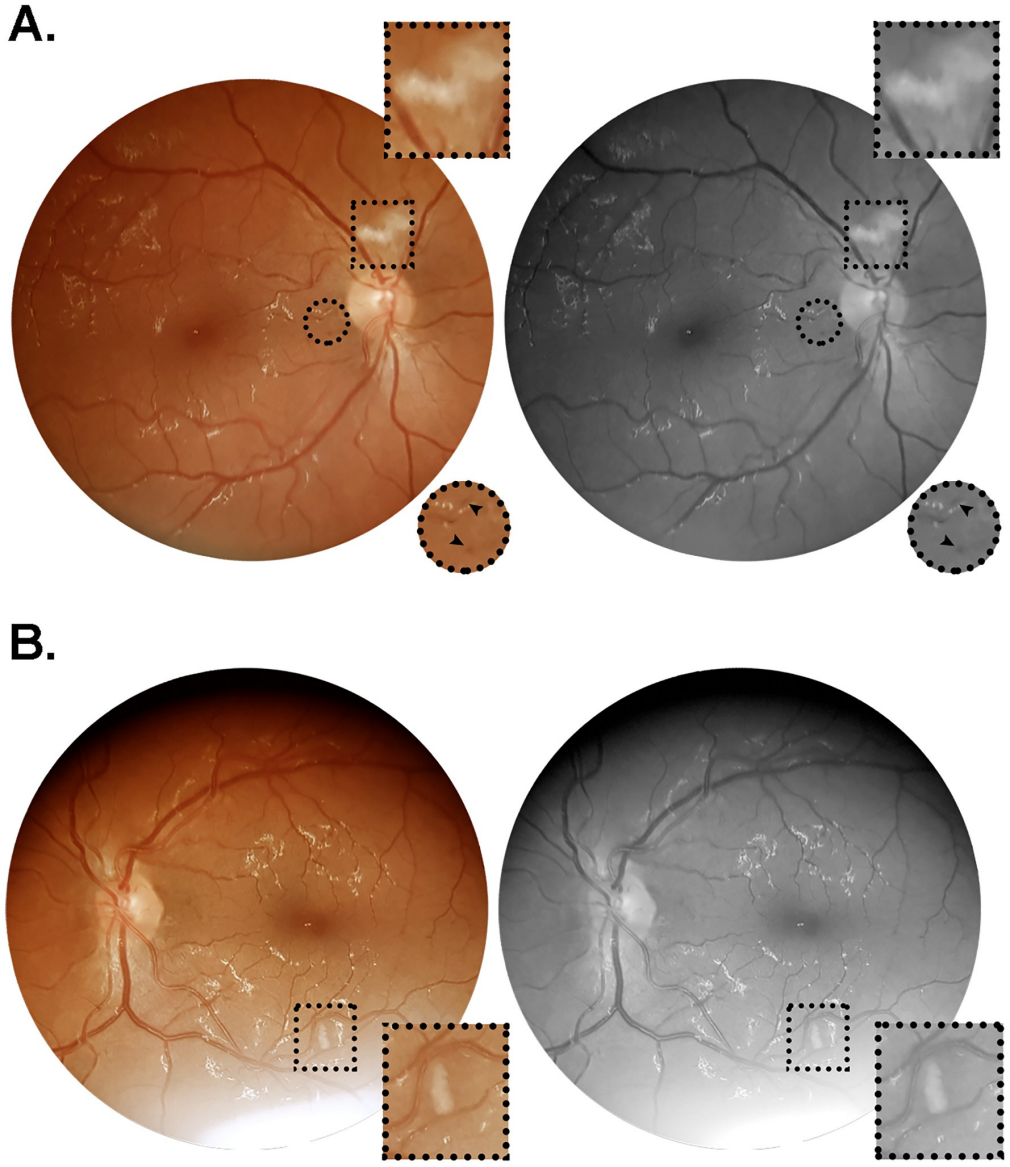
Color and red-free retinal photographs from patient #6 (W.A.C.). Images acquired on day 64 after symptoms onset, from a 35 year-old obese and hypertensive man who was admitted on day seven since onset of fever, cough and dyspnea. During hospitalization, this patient evolved to critical illness, characterized by acute respiratory distress syndrome, respiratory failure and acute kidney injury, requiring ICU monitorization, mechanical ventilation and hemodialysis. The patient had a positive clinical outcome, and received hospital discharge on day 69 after symptoms onset. (A) Right retina showing a nerve fiber layer infarct above the optic nerve head, and microhemorrhages in the papillomacular bundle close to the optic disc. (B) Left retina showing nerve fiber layer infarcts at the inferior temporal vascular arcade, approximately 1.5 disc diameters inferior to the macula. Square and circular insets represent 2x magnification of the delimited area.

Patient #6 was a 35 year-old hypertensive and obese man, who presented ground-glass peripheral opacities occupying 25–50% of the organ parenchyma at admission lung CT scan, progressing 15 days later to 50%. His blood parameters on admission were CRP 6.1 mg/dL, D-dimer 0.27 mcg/mL (Table 2). Retinal examination was performed on day 59 post symptom onset, showing nerve fiber layer infarcts in both eyes, adjacent to the optic nerve head in the right eye (Fig 1A) and close to the inferior temporal vasculature in the left (Fig 1B). Upon reexamination 5 days later (day 64 shown in Fig 1), there were no changes in terms of intensity or extension of the lesions previously observed. Additionally, discrete microhemorrhages were noticed in the papillomacular bundle in both eyes. He had a critical phenotype of COVID-19, requiring endotracheal intubation and mechanical ventilation in the ICU due to acute respiratory distress syndrome. During the hospital stay, this patient developed hemodynamic instability, with multiple episodes of systemic hypotension requiring continuous catecholamine infusion at high doses, associated with acute renal injury requiring hemodialysis. Moreover, severe and symptomatic anemia was observed, with hemoglobin levels and hematocrits dipping to values below 6.5 g/dL and 21.0%, respectively. Dysesthesia plus flaccid weakness were evident on distal lower and upper limbs, characterized as sensory and motor axonal polyneuropathy on electroneuromyography test and diagnosed as a neuropathy of critical illness by the medical team (Table 2). When ophthalmological examination was performed, this patient was clinically stabilized with normal kidney function.

**Fig 2:**
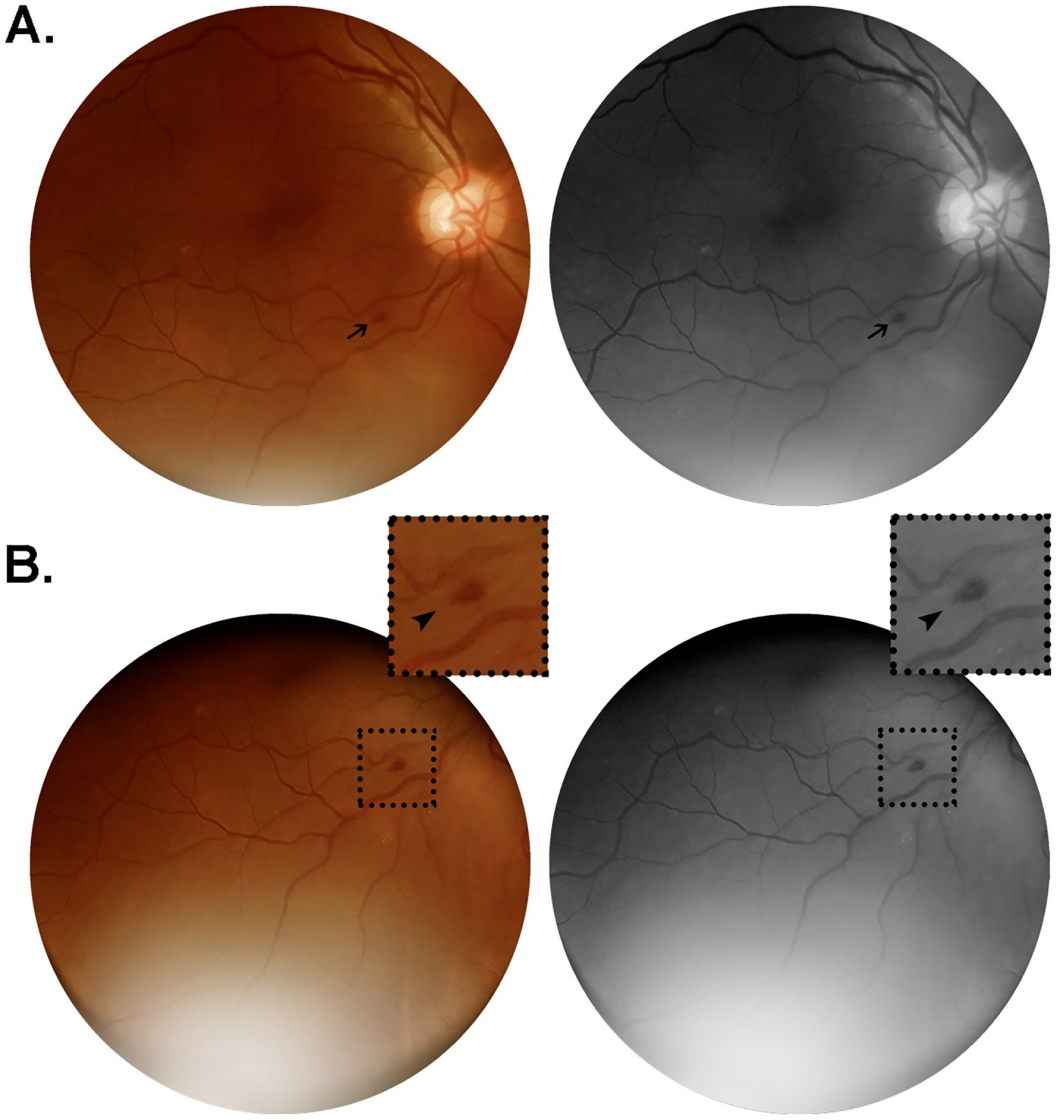
Color and red-free retinal photographs from patient #18 (W.S.). Images acquired on day 16 from symptoms onset, from a 56 year-old man with no comorbidities, admitted on day 13 after onset of a clinical scenario characterized by fever, cough, headache, myalgia and dyspnea. The patient evolved to severe illness, not requiring ICU monitorization nor developing any organ failure. Lung CT at admission revealed bilateral ground-glass opacities predominantly in the periphery, extending to approximately 50% of lung parenchyma. (A) Flame-shaped hemorrhage (arrow) approximately one disc diameter from the optic nerve head, close to the inferior temporal vascular arcade. (B) Same lesion observed from an alternative point of gaze fixation, magnified in the insets (2x; arrowheads).

Patient #18 (Table 2) was a 56 year-old man referred in with no comorbidities. He presented with a unilateral flame-shaped lesion approximately one disc diameter from the optic nerve head, adjacent to the inferior temporal vascular arcade (Fig 2). The contralateral retina had no vascular lesions observed. This patient was admitted 13 days after onset of symptoms (fever, cough, headache, myalgia and dyspnea), had 50% ground-glass opacities bilaterally on pulmonary parenchyma (chest CT scan), and evolved to a severe phenotype but not requiring ICU continuing monitorization or mechanical ventilation. Fundus examination was performed on day 16 after symptoms onset (Table 2). Blood parameters on admission were CRP 12.1 mg/dL, D-dimer 5.92 mcg/mL and ferritin 1030.0 ng/mL (Table 2). Due to particularly high levels of D-dimer during hospitalization, this patient received full anticoagulation with heparin instead of the prophylactic dose routinely used for COVID-19 inpatients.

**Fig 3:**
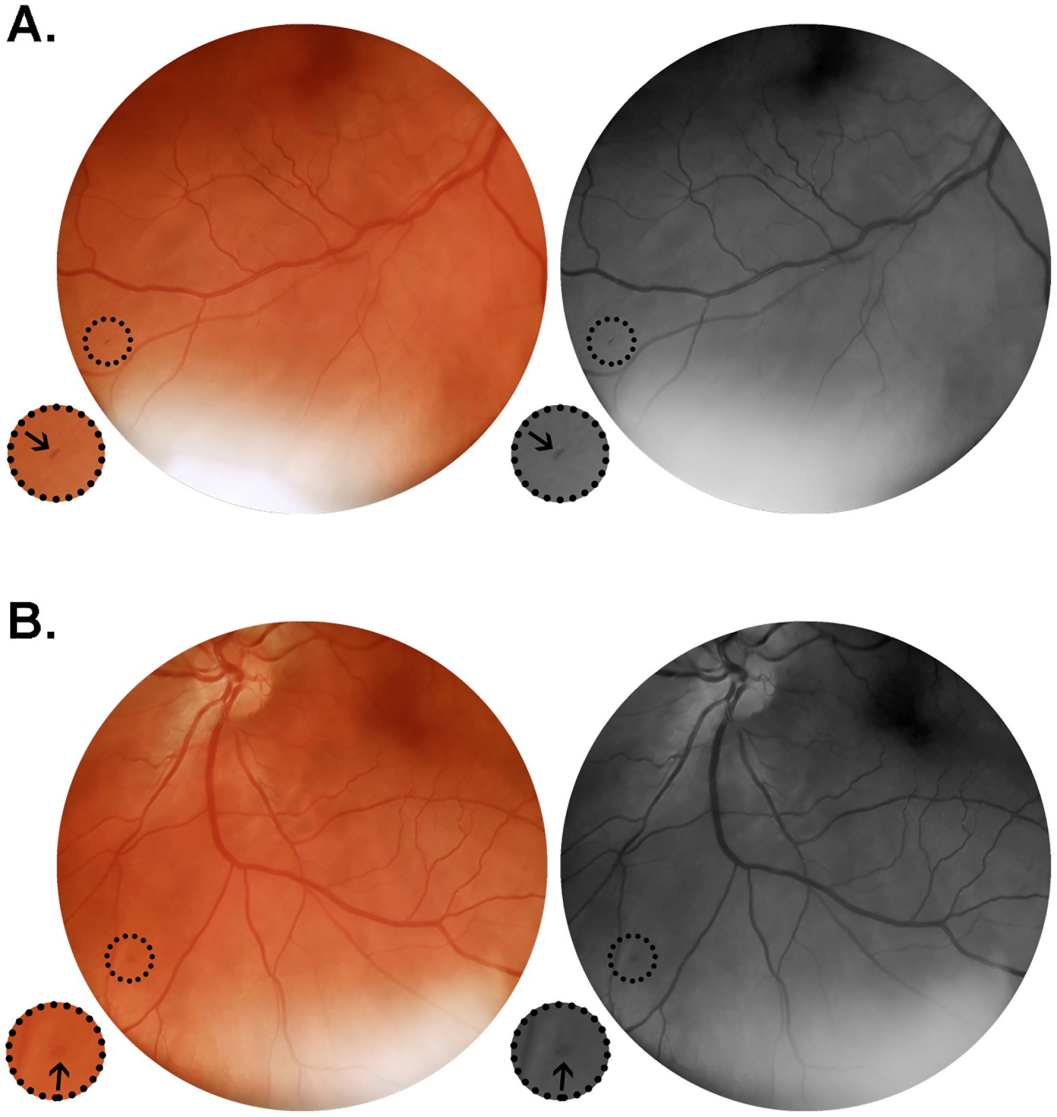
Color and red-free retinal photographs from patient #19 (G.C.X.). Images acquired on day 12 from symptoms onset, from a 49 year-old man in regular treatment for arterial hypertension, admitted on the eight day after onset of fever, cough, myalgia and progressive dyspnea. Lung CT on admission showed typical viral pneumonia findings extending through 25–50% of the parenchyma. The patient evolved to a severe phenotype of the disease. (A) Right retina presenting with isolated microhemorrhage at the periphery close to the inferior temporal vasculature. (B) Left retina showing isolated microhemorrhage at the inferior nasal retinal quadrant. Insets represent delimited areas with 2x magnification.

Patient #19 was a 49 year-old hypertensive man hospitalized on the eighth day after symptoms onset (fever, myalgia and dyspnea). He presented with isolated microhemorrhages in both eyes in the inferior retinal quadrant close to the vascular arcades (Fig 3). Retinal documentation was done on day 12 of symptoms onset. This patient, admitted with altered blood parameters (CRP 6.8 mg/dL; ferritin 1774.0 ng/mL) and abnormal lung CT scan (25–50% ground-glass opacities extension), evolved to a severe COVID-19 phenotype, but not requiring ICU monitorization or mechanical ventilation (Table 2).

None of the other 22 patients enrolled demonstrated overt retinal changes upon careful analysis of both color and red free retinal photographs.

## Discussion

Surprisingly, of the 25 patients with severe or critical disease in our study, only three (12%) manifested convincing retinal changes (microhemorrhages, flame-shaped hemorrhage and nerve fiber layer infarcts). Of their six eyes, five showed retinal lesions. These three patients were in late phases of COVID-19 disease (#6: 59 and 64 days of symptoms onset; #18: 16 days; #19: 12 days). As retinal lesions as described in the present study are not uncommon in septic patients with infectious diseases [19], our results underline that comorbidities, clinical intercurrences or the robust immune response are more likely related to the retinal findings, instead of a direct neuronal injury from SARS-CoV-2 as previously suggested in mild to moderate COVID-19 outpatients [20]. Patient #6, for instances, was critical and had severe anemia and hypotension, as well as injury to other organs including kidneys and the peripheral nervous system, which could justify the bilateral nerve fiber layer infarcts and microhemorrhages in the papillomacular bundle found upon dilated retinal examination. Patient #18 presented with high serum levels of D-dimer (>5.0 mcg/mL) and required full dosing of subcutaneous enoxaparin for anticoagulation leading to higher bleeding risk; associated with intermittent episodes of cough (Valsalva maneuver), one of the patient’s symptoms (Table 2), this could be related with the isolated unilateral flame-shaped hemorrhage shown upon retinal examination. Patient #19, on the other hand, had systemic hypertension, and although hypertensive retinopathy was not apparent, intermittent blood pressure peaks occurred during his hospital stay and there was a likely fragile blood-retinal barrier due to systemic inflammation, as reflected by elevated inflammatory biomarkers on admission (CRP: 6.8 mg/dL; ferritin: 1774 ng/mL) (Table 2). Genetic factors may have also influenced the emergence of retinal abnormalities in the three patients depicted in the presented study, in contrast to the other 22 patients who did not present any retinal changes upon careful retinographies evaluation, despite the severe or critical disease phenotype with varied related clinical intercurrences during hospitalization. On the other hand, disease phenotype, per se, does not seem to impact the findings on fundus examination in the present study, as comparison between severe and critical patients showed no statistical significance in terms of retinal changes (Table 1).

Severe pneumonia is the major life-threatening clinical manifestation of COVID-19. This may lead to respiratory distress and lung failure. However, extrapulmonary sites of the disease have been recognized. Various distinct types of neurologic involvements have been reported, such as cerebrovascular diseases, encephalitis, acute necrotizing encephalopathy, meningitis/encephalitis, olfactory and gustatory disorders independent of nasal mucosa inflammation, demyelinating neuropathy (e.g. Guillain-Barré syndrome), cranial polyneuropathies (e.g. Miller Fischer), radiculopathy and large intracerebral arteria wall inflammation [21–25]. Some of these may relate to parainfectious processes, hypoxic injury or dysfunctional immune responses mediated by numerous immunomodulatory molecules like IL-6, IP-10, IFN γ, IL-2, IL-10, G-CSF, MIP1α, and TNF (widespread “cytokine storm”) [26, 27]. In fact, the inflammatory cytokine storm that marks the later stage of the disease in severe or critically ill patients, together with hypercoagulable state and decreased arterial blood oxygen tension, are closely related to the development of multiorgan damage including acute respiratory distress syndrome (ARDS), renal failure and cerebrovascular disease [28], and could also be associated with the retinal findings described in our study [29].

Importantly, the identification of viral RNA in cerebrospinal fluid in case reports [30] along with the presence of SARS-COV-2 spike protein receptor ACE2 in the central nervous system, particularly in vascular endothelium [9], requires us to consider the hypothesis of SARS-CoV-2 directly damaging CNS structures, including the retina. As well, an alternative host cell entry route through the receptor CD147 has been characterized [31], and a moderate to high expression of CD147 gene has been shown in human retina [32]. Moreover, positive RT-PCR has been reported in human retinal biopsies, suggesting that the viral genome may be directly infecting retinal neurons [15], and in other vertebrate species, specific coronavirus strains are able to infect retinal neurons both *in vitro* in retinal cell cultures [33] and *in vivo* after intravitreal inoculation of viral particles [34], causing a retinal degeneration named experimental coronavirus retinopathy (ECOR) [35, 36]. This has been shown to be a useful tool to experimentally model human progressive degenerations affecting photoreceptors and retinal pigment epithelium (RPE) [34]. In felines, coronavirus (feline infectious peritonitis virus or FIPV) is highly prevalent in the conjunctiva of infected animals, and may be associated with ophthalmological manifestations such as conjunctivitis, anterior uveitis, choroiditis with retinal detachment and retinal vasculitis [37].

A recent study specifically investigated neurologic involvement in hospitalized patients, noting that one-quarter of confirmed COVID-19 individuals had some manifestations of CNS affection [38]. Visual impairment was mentioned in 3% of patients, 2% being severe; The presence or absence of retinal changes was not documented.

Retinal lesions have been reported in outpatients after confirmed SARS-CoV-2 infection with mild to moderate symptoms, some of them characterized by non-specific and controversial hyper-reflective optical coherence tomography (OCT) lesions in the ganglion cell and inner plexiform layers, microhemorrhages and nerve fiber infarcts [20, 39], in addition to another report of two patients showing OCT lesions suggestive of paracentral acute middle maculopathy (PAMM) and acute macular neuroretinopathy (AMN), although a true association between the retinal pathologies and COVID-19 could not be made [40]. More recently, evaluation of 27 outpatients with retinal fundoscopic, B-scan OCT and OCT angiography at mean of 43 days after COVID-19 symptoms onset showed retinal microangiopathy manifested as cotton wool spots (clinical sign associated with nerve fiber infarcts) in 22% of patients [41].

Laboratorial parameters from blood samples have been shown in COVID-19 patients, such as D-dimer, ferritin and C-reactive protein. These were elevated in most of the patients in our study (Table 2). Hyperferritinemia is associated with several inflammatory conditions, such as sepsis, systemic inflammatory response syndrome (SIRS), multiorgan dysfunction syndrome (MODS), and macrophage activation syndrome (MAS). In critically ill patients, hyperferritinemia is associated with the severity of the underlying disease [42]. Elevation of D-dimer underlines an altered coagulation system with cascade dysregulation leading to thrombotic events mostly associated with poor prognosis in COVID-19 [6]. Blood CRP and D-dimer have been shown to be significantly higher in severe COVID-19 patients when compared to non-severe cases [38]. In our study, there was no difference between groups (severe vs critical) on admission ferritin, D-dimer or CRP values (Table 2). Nevertheless, we need to study a larger number of patients to gain sufficient statistical power for such comparisons [38].

A major strength of our study was the bedside retina documentation from confirmed COVID-19 patients still hospitalized for clinical management of severe and critical disease. Upon retina examination, patients were mostly in the inflammatory phase and so more likely to present with systemic hypercoagulopathy, cytokine storm and blood clots formation in small and large arterial vessels and in the venous system, leading to infarcts in several tissues throughout the body, including myocardium, kidneys and lungs. In the CNS, acute cerebrovascular events, both ischemic or hemorrhagic, have been described in COVID-19 patients, some of them reported as subclinical findings or even asymptomatic.

As a major limitation of this study, this was a small case series involving 47 eyes from 25 patients. In addition, retinal photography is not sensitive enough to detect small arteriolar or capillary changes in the retinal circulation, nor to demonstrate discrete histological changes in individual retinal layers. OCT, OCT-angiography, fluorescein angiography or electroretinogram (ERG) may reveal subtle lesions. However, these are difficult tests that require much instrumentation and their use in still hospitalized inpatients with droplet precaution is problematic and largely infeasible. Another limitation is a likely underestimation of the number of retinal findings as three patients from this series had the retinographies of only one eye evaluated: #13 and #25 did not consent to undergo pupil dilation, which lead to low image qualities in one of the eyes due small pupil diameter; and #24, who at the time of retinal examination was in the pre-operative period for subarachnoid hemorrhage and had highly anisocoric pupils, so the examiner was advised by the neurosurgery team to not induce pharmacological mydriasis in the miotic eye.

## Conclusion

To the best of our knowledge, our study is the first to show that funduscopically evident retinal vascular lesions such as flame-shaped or microhemorrhages and nerve fiber layer infarcts may be present in not insignificant percentage of severe or critically ill COVID-19 patients during their hospitalization. These retinal changes, only seen after morbid developments, were likely secondary to clinical intercurrences or comorbidities, and may be important and easily accessible outcome measures of therapeutic interventions and sentinels of neurologic and systemic diseases during COVID-19 pandemic.
